# The Health of London

**Published:** 1854-10

**Authors:** 


" *The Health of London.*—The weekly report on the health of London, published by the authority of the registrar-general, states that the number of deaths from all causes returned for the week that ended Saturday the 19th of August, was 1,833, that of the previous week was 1,832. In the ten weeks corresponding to last week, of the years 1844-53, the average number was 1,113, which, if raised in proportion to increase of population, becomes 1,224. The present return exhibits an excess of 609 above the estimated amount.

" Cholera was fatal last week to 729 persons, of whom 214 were children under 15 years of age, 426 were 15 years of age and under 60, and 88 were 60 years old and upward. During the cholera epidemic of 1849 the total deaths registered in the week that ended August 18, were 2,230, and those from cholera were 1,230. In the six weeks of its present appearance, the deaths from cholera have been successively 5, 26, 133, 399, 644, and 729. The deaths from diarrhœa last week were 192.

" In comparing the mortality from cholera in London with the deaths from the disease in other places, the population and vast extent of the metropolis must be taken into account.

"The subjoined figures show that cholera has prevailed with great irregularity over London, and that in several sub-districts the ravages of the epidemic are inconsiderable. Imperfect drainage, proximity to the dirtiest parts of the Thames, bad water, and poverty are still, as they were in 1849, the chief circumstances that make cholera fatal. It is on the banks of the polluted Thames, in the lower parts of the London basin, that the people die in large numbers; for on ground not on an average ten feet above the Trinity high-water mark, 1,212 of the deaths from cholera have happened out of 595,119 people, while in the next terrace of ten feet, and under forty feet of elevation, 493 in 648,619 have died; and on the higher grounds above

St. James's-square and the Strand, only 213 have died of cholera out of 1,070,372 inhabitants. The mortality at the three elevations commencing at the lowest, has been at the rate of 204 and 76 and 20 to every 100,000 inhabitants. The people on the low grounds have suffered ten times as much as the people living on the grounds of a moderate elevation.

"The 729 deaths from cholera in the week now reported were distributed according to districts thus: West Districts, elevation above Trinity high-water mark, 28 feet; population in 1851, 376,427; deaths, 184. North districts, elevation, 135 feet; population, 490,386; deaths, 38. Central districts, elevation, 49 feet; population, 393,296; deaths, 32. East districts, elevation, 26 feet; population, 485,522; deaths, 105. South districts, elevation, 6 feet; population, 616,635; deaths, 370.

"Last week the births of 813 boys, and 756 girls, in all 1,509 children, were registered in London. The average number in nine corresponding weeks of the years 1845-53 was 1,363.

"At the royal observatory, Greenwich, the mean height of the barometer last week was 29.813 in. The mean reading on Friday was 30.022 in. The mean temperature of the week was 59.9 deg. which is 1.2 deg. below the average of the same week in 38 years. The mean daily temperature was below the average, except on Sunday, Monday, and Saturday. It was so much as 6.4 deg. below it on Thursday. The highest temperature of the week occurred on Sunday, and was 79.8 deg.; the lowest was 43 deg. on Friday. The mean dew-point temperature was 51.2 deg., and the difference between this and the mean temperature of the air was 8.7 deg. The temperature of the water of the Thames rose to 66 deg. on five days of the week. The wind blew generally from the south-west. The whole amount of rain was 0.15 in."

A friend has handed us the foregoing, cut from "Lloyd's Newspaper," (London,) of a recent date. We have before called attention to the influence of altitude in determining the severity of epidemics, as stated by the registrar-general of England. The exact means of sanitary survey in London, and the ease with which they can ascertain the population of a given district, confer a peculiar value upon the sanitary statistics of that great city.

---

For the Buffalo Medical Journal.

Most of the physicians of our country have had occasion to complain of the parsimonious and oppressive disposition manifested by the superintendents of the poor, in allowing pay for services rendered to the public in their care of the sick poor. Very few, if any, have had the honor of presenting a bill to that board, accompanied with their solemn oath that it was correct, but have had those same bills cut down from 25 to 75 per cent. The same disposition has been manifested in procuring medical services for our public institutions: the salary being merely nominal, while the services were often really responsible and onerous. For this latter, however, physicians were themselves, perhaps, more to blame, as in their anxiety for the honors and drippings of office they would underbid each other to an almost ruinous extent.

These evils had grown to such magnitude that the profession felt it was necessary to agree on some combined action to secure their own rights without infringing upon those of the public.

At the semi-annual session of the county Medical Society in June last, a committee which had been appointed for that purpose at the annual meeting in January previous, brought in a report, which after a free interchange of opinion, was adopted with great unanimity, if not unanimously. Among other items the minimum salary of physician to the Almshouse was fixed at $600 per annum; and it was agreed to consider it dishonorable to accept the place at a less sum. No one expressed an opinion that this minimum was fixed too high—indeed most (and the attendance was unusually large) considered $1000 as the more appropriate figure, but as a measure of expediency it was thought advisable to agree upon the first mentioned sum.

So far so good. And now for the sequel. The term of service of the present physician to the Poorhouse expires on the 1st prox.; the Board of Superintendents met, for the purpose of appointing a successor, on the 19th inst. Several applications for the soon vacant post were before them. After examining the various petitions, proposals, recommendations, &c., they discovered a chance to save a few dollars to the county, as one man who happens to have a Dr. prefixed to his name, more anxious for the dollars than his honor—fearing the consequences of an equal contest, and doubtless estimating his services at their full value—proposed to perform the duties of the office for $400, and the Board thinking, perhaps, he *would do well enough for the poor*, accepted. If "every man has his price," it is, perhaps, fortunate that our brother has fixed his, so that others may not overestimate it.

Doubtless the disappointed applicants feel somewhat sore at this result—but they should remember that cheap wares are soonest sold, especially if possessed of a flashy exterior. Now then, editors, as you profess to be conservators of medical ethics and etiquette, in what light should such a transaction be viewed by the profession? Can one who thus barters the dignity of his profession and manhood, and throws contempt on the society of which he is a member, claim the courtesies of his outraged brethren? Pray tell me of what use are our societies, if the members are not to be bound by their decisions? So long as physicians can, under such circumstances, underbid their competitors with impunity, what chance of success have they who still think there is such a thing as honor yet in the world?

Sept. 20, 1854. JUSTICE.

Let our correspondent be consoled.

"Cheap and nasty" is as true in physic as in trade; and nowhere have we witnessed a better exemplification of that time-honored maxim, than in the instance which excites his ire.

Let "Justice" keep cool. The poorhouse and its management is a "visitation of God," and the philanthropic may as well shut their eyes and submit. H.

